# Analysis of Gender-Based Inequality in Cardiovascular Health: An Umbrella Review

**DOI:** 10.7759/cureus.43482

**Published:** 2023-08-14

**Authors:** Jodie Bosomworth, Zahid Khan

**Affiliations:** 1 Cardiac/Thoracic/Vascular Surgery, West Suffolk Community Cardiac Rehabilitation Team, West Suffolk NHS Foundation Trust, Suffolk, GBR; 2 Acute Medicine, Mid and South Essex NHS Foundation Trust, Southend on Sea, GBR; 3 Cardiology, Bart’s Heart Centre UK, London, GBR; 4 Cardiology and General Medicine, Barking, Havering and Redbridge University Hospitals NHS Trust, London, GBR; 5 Cardiology, Royal Free Hospital, London, GBR

**Keywords:** gender inequalities in treatment, post-menopause, poor assessment / misdiagnosis, prisma style flow chart, pico format, systematic reviews and meta-analyses, scad and takotsubo cardiomyopathy., cvd risk assessment, gender-based inequality in cardiovascular health, an umbrella review

## Abstract

This umbrella review aims to investigate possible gender-based inequality in cardiovascular health and improve understanding surrounding differing presentations seen in women. Searches of current literature were conducted using Medline; Cochrane; Cumulative Index of Nursing and Allied Health Literature (CINAHL Plus); and PubMed databases. Focusing on systematic reviews and meta-analyses from the last decade, searches were expanded to the publication year of 2000 onwards, to enable a broader review of current practices. Current clinical guidelines were also reviewed. 17 articles were deemed to satisfy the desired criteria and were therefore carried forward to be critically appraised. The articles reviewed were multifactorial; however, they can be grouped into four categories of common focus: risk factors, presentation, treatment, and current research. On critical analysis, 13 reoccurring themes were noted throughout the reviewed articles with each discussed in detail within this review. There is a need to prioritize women in cardiovascular health, through raising awareness, improving prevention (both primary and secondary), reducing delays in presentation, and increasing understanding and recognition of sex differences in symptom presentation, to enable improved diagnosis and treatment along with the standardization of gender-specific clinical guidance. The results are unanimous regarding an undeniable gender-based inequality in cardiovascular health to the detriment of women. With such damning evidence that women are under-represented and indeed undertreated, the time has come now to question whether women should be considered as their own specialty within cardiology and to ultimately raise awareness and ensure women are given the same consideration regarding cardiovascular disease (CVD) risk assessment and treatment, in order to finally remove gender inequality in cardiovascular (CV) health. In order to reverse this disparity, it is clear from the included studies that further research is required to understand the sex differences seen in both the presentation and symptoms of CVD as well as to enable improved treatment of women and the development of sex-specific strategies and clinical guidance to empower clinicians moving forward.

## Introduction and background

On undertaking this project, the sheer void in knowledge and understanding with regard to women and cardiovascular disease (CVD) became increasingly obvious and was sadly echoed from paper to paper [1-23]. The travesty of inequality in cardiovascular (CV) health is clearly to the detriment of women.

CVD remains the leading cause of death among women globally, being attributable to 35% of deaths in women in 2019 [1]. However, there remains a misconception that women are at a lower risk of CVD than men. Although women defer risk by 10 years, they do not avoid it, with higher rates of mortality attributed to CVD in women than men [2]. The investigator believes there is currently a lack of individualization in the recognition, intervention, and rehabilitation of women in cardiology. With emerging conditions such as spontaneous coronary artery dissection (SCAD) predominantly found in women, a better understanding is needed. It is time for healthcare professionals to recognize that the “old” one-size-fits-all approach is no longer acceptable, especially when delays in diagnosis and treatment are a common occurrence for women as their presentations do not fit with old ideals or traditional symptoms of myocardial infarction (MI) and other cardiac conditions.

Thompson and Daugherty (2017) note that CVD historically is seen as a “man’s disease”, despite campaigns to increase awareness, the sad fact remains that the number of women diagnosed with and dying from CVD is ever-increasing worldwide [3].

Thompson and Daugherty acknowledge that biological differences across genders lead to varying outcomes, however, they attribute disparity in CV care as the likely culprit with inadequate screening, delayed diagnosis, and/or poor treatment. Knowledge-mediated bias can be caused by assumptions made by clinicians, leading to particularly younger women <54 years being assumed low risk of CVD and therefore rarely prescribed preventative therapy [3].

Zhao et al. (2020) completed a meta-analysis to review gender-based differences in the prescription of CVD therapy in primary care and concluded that women with an established high-risk status or diagnosis of CVD were less likely to be prescribed aspirin, statins, or angiotensin-converting enzyme inhibitor (ACEi), whereas there was a lower prevalence of prescribed diuretics in men [4]. Zhao et al., acknowledge the need for further research into the reasoning for such disparities and for strategies to be made to address them [4].

Mitchell (2021) eloquently summarises the thoughts of many experts in this field “CVD among women remains understudied, under-recognized, underdiagnosed, and undertreated, with women under-represented in clinical trials” [5].

With such damning evidence that women are under-represented and indeed undertreated, then the time has come to question whether women should be considered as their own specialty within cardiology, to ultimately raise awareness and ensure women are given the same consideration regarding CVD risk assessment and treatment, in order to finally remove gender inequality in CV health.

## Review

Aims

The aim of this research is to investigate possible gender-based inequality in CV health and improve understanding surrounding differing presentations seen in women. Key to this research is highlighting differing presentations within CVD such as those often described following MI, and to examine our emerging understanding of conditions such as SCAD and takotsubo cardiomyopathy. Further understanding will also be sought through a thorough examination of gender-based differences in risk factors such as lipid profiles, and hormones (pre and post-menopause).

Methodology

This project was conducted as an umbrella review to best meet the proposed aims, therefore focusing on the highest level of current evidence available in this field with the utilization of current systematic reviews (SR) and meta-analyses (MA). This has enabled the investigator opportunity to focus on reviewing the current best evidence-based medicine available to healthcare professionals regarding the prevention, diagnosis, and treatment of women with CVD.

Inclusion Criteria

The following inclusion criteria were followed using the population, intervention, control, and outcomes (PICO) format:

Population: Data was included from multiple participants over eighteen years from all demographic groups.

Interventions / phenomena of Interest: The aim of the project is to focus on gender-based inequalities in CV health, therefore incorporating prevention, diagnosis, and treatment.

Context / setting: As noted, particular focus is placed on women across all healthcare settings.

Outcomes: The key element of this study was to investigate any gender inequalities within CV health, with the aim to highlight any gender differences recognized in differing presentations (particularly: myocardial infarction (MI), SCAD, and takotsubo), risk factors, or treatments, which could lead to changes in practice, improving outcomes for women with CVD.

Searches of current literature were conducted through the following library databases: Medline; Cochrane; CINAHL Plus; and PubMed, with a focus on systematic reviews and meta-analyses along with current clinical guidelines from the last decade. Searches were further expanded to the publication year of 2000 onwards, to enable a broader review of current practices. The search was limited to English as the language of publication with a primary focus on data originating from Western countries. The investigator chose to exclude studies from non-Western countries to enable a particular focus and comparison of current clinical guidance from the National Institute for Health and Care Excellence (NICE) and the European Society of Cardiology (ESC) as discussed later in this proposal. The literature within this report has also been supplemented using Google Scholar.

Please refer to Table 1 below for a summary of the key terms and concepts used within the literature search to meet the outlined aims for the project, along with the inclusion and exclusion criteria.

**Table 1:** Summary of the key terms and concepts used within the literature

Variables	Criteria	
	Inclusion	Exclusion
Terms and Concepts	Gender / Sex / Women And Inequalities / Disparities / Bias And Cardiovascular Disease / CVD / Cardiac / Heart Health With a particular focus on the following key areas of interest for inclusion: Risk Factors / Hormones / Lipids Menopause Or MI / SCAD / Takotsubo Prevention / Diagnosis / Treatment	
Publication Types	Systematic Reviews (SR) Meta-analyses (MA) Clinical Guidelines	Reviews of theoretical studies or published opinions as primary sources were automatically excluded.
Databases	Medline Cochrane CINAHL Plus PubMed Supplemented by Google Scholar	
Language of Publication	English	Non-English Language
Year of Publication	Primary focus on 2010 onwards 2000 onwards	Prior to the year 2000
Age Group	Over 18 years	Under 18 years
Country of Origin	Western Countries	Non-Western Countries

**Figure 1:**
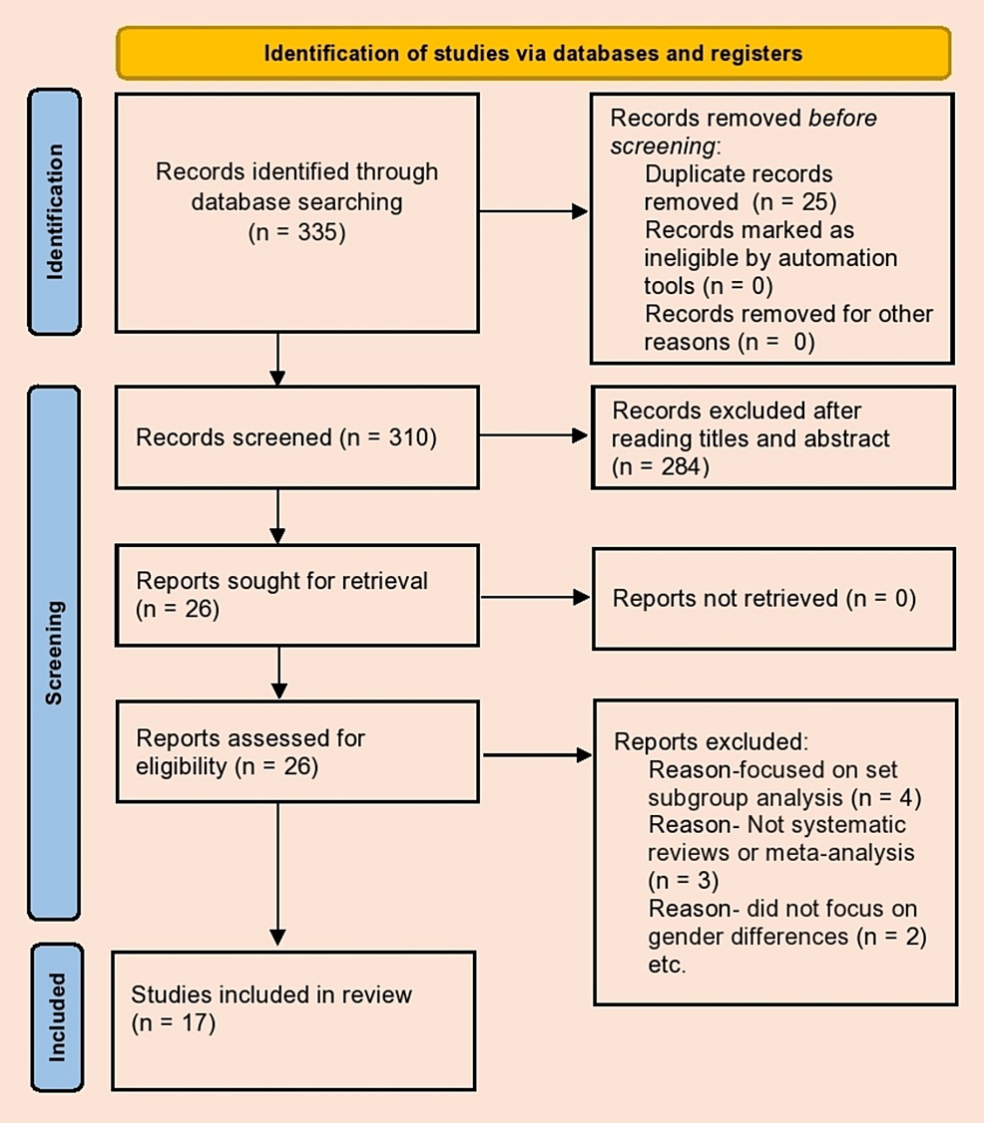
PRISMA 2020 flow diagram Preferred Reporting Items for Systematic Reviews and Meta-Analyses (PRISMA)

On completion of the search, 335 articles were identified related to this study. A total of 309 articles were excluded after reviewing their titles and abstracts and removing duplicates. Only 26 articles were screened in full and a further nine articles were removed for not meeting the inclusion criteria (Figure 1). Nine articles were excluded as they did not fulfil the set inclusion criteria; of these, the methodology of three articles did not match (did not conduct a systematic review or meta-analysis), four articles focused on subgroups only, and two did not focus on gender differences. Thus, 17 articles were considered to satisfy the desired criteria and were therefore carried forward to be critically appraised. Please refer to Figure 1 for a complete breakdown shown in a Preferred Reporting Items for Systematic Reviews and Meta-Analyses (PRISMA) flow chart.

It is prudent to highlight that one of the limitations of this umbrella review is the fact that appraisal of all included literature has been undertaken by the independent reviewer. To reduce possible bias, data from each literature review was critically appraised using a set checklist which included reviewing the characteristics of each study [6]. The critical review has also been supported using the Grading of Recommendations Assessment, Development and Evaluation (GRADE) checklist, assessing the quality of the systematic reviews included within this umbrella review. The GRADE checklist was chosen as it is a reproducible and transparent framework, with the flexibility to adjust the certainty in evidence at the discretion of the reviewer [7].

Supplementary information has also been obtained to support and elaborate on key findings from the umbrella review through other sources such as pertinent books, patient information, and websites. Later within this umbrella review the current clinical guidance available to clinicians has also been thoroughly reviewed, with aspirations for the future regarding women and CV health discussed.

Literature review

In the following section, the investigator plans to discuss the key points and reoccurring themes noted within the articles included in this review. It is also important to recognise the broad variety of studies included within this review, all consisting of variable methods and research types, with multiple varying hypotheses. There is an undeniable significant level of heterogeneity within this review; therefore, no formal meta-analytic techniques have been undertaken. High levels of heterogeneity were also commonly acknowledged by authors across all studies included.

Results

As noted, Table 2 summarises the aims of each paper reviewed. The grade level and percentage of female representation within each study has also been highlighted. Some key results from the included studies are highlighted in Table 2. However, the investigator has chosen not to focus on these in response to the set question of the umbrella review, choosing to focus in further detail on the reoccurring themes found across the included articles which are deemed more relevant to the set aims of this project; these can be found in the discussion section.

**Table 2:** Summary of the included article characteristics Cardiovascular disease (CVD), socioeconomic status (SES), outpatient cardiac rehab (OCR), chronic total occlusion (CTO), ST elevated myocardial infarction (STEMI), primary percutaneous coronary intervention (PPCI), acute coronary syndrome (ACS), non-ST elevated myocardial infarction (NSTEMI),

Article	Authors	Year	Study Type	Representation of Women within Study	Number of Studies Included	Objective of Study	GRADE Score
1	Zhao et al. [4]	2020	Systematic Review & Meta-Analysis	27.8%	43	To investigate sex differences in cardiovascular medication prescription among patients at high risk or with established CVD in primary care.	High
2	Backholer et al. [8]	2017	Systematic Review & Meta-Analysis	35%	116	The focus of this study was reviewing whether the impact of socioeconomic status (SES) on men and women was comparable in terms of CVD risk.	High
3	Caceres et al. [9]	2017	Systematic Review	Unclear	31	Highlighted gap in research regarding CVD among sexual minority adults. Institute of Medicine identified CVD research on sexual minorities as an area with an urgent need for further research, therefore the aim of this study was to synthesise and critique the existing evidence from studies that compared CVD risk factors and CVD diagnoses between Sexual Minority and Heterosexual Adults.	Moderate
4	Colella et al. [10]	2015	Meta-Analysis	33.5%	19	Aim of the study to review studies published in the last 10 years that have examined differences in Outpatient Cardiac Rehab (OCR) referral rates of men and women to assess if sex differences still exist. Referral rates remain low despite clear benefits being well documented.	High
5	Giorgini et al. [11]	2017	Meta-Analysis	Unclear	10	Aim of the study to evaluate all available data exploring sex and gender differences in clinical outcomes in hypertensive patients under treatment in order to quantify the impact of gender in outcomes associated with the antihypertensive treatment.	High
6	Hamill and Ingram [12]	2015	Systematic Review	Unclear	33	To evaluate and summarise the current evidence base for gender-specific care in relation to coronary heart disease.	High
7	Lewey et al. [13]	2013	Meta-Analysis	54.2%	53	To evaluate the effect of gender and race on adherence to statin therapy in primary or secondary prevention.	High
8	Mannem et al. [14]	2019	Systematic Review & Meta-Analysis	27%	9	To evaluate possible sex differences and outcomes after percutaneous intervention in patients with chronic total occlusion (CTO).	Moderate
9	Meads et al. [15]	2018	Systematic Review & Meta-Analysis	Unclear	16	Evaluation of CVD, hypertension, respiratory disease, and diabetes in sexual minority women.	Moderate to High
10	Nguyen et al. [16]	2010	Systematic Review	20 - 50%	44	Review of literature examining age and sex differences with regards to the pre-hospital delay in patients hospitalized with acute myocardial infarction.	Moderate to High
11	Oertelt-Prigione et al. [17]	2011	Systematic Review	Unclear	405	To investigate the frequency and type of published gender-specific research in vascular diseases, including the type of study, funding, country of origin, and gender of the author.	Moderate
12	Pancholy et al. [18]	2014	Meta-Analysis	27%	35	Examination of differences in mortality by sex in patients with ST elevated myocardial infarction (STEMI)I treated with primary percutaneous coronary intervention (PPCI).	High
13	Panza et al. [19]	2019	Systematic Review	-	84	Review examining evidence linking discrimination and cardiovascular health among socially stigmatized groups.	Low
14	Shah et al. [20]	2021	Meta-Analysis	31%	56	To increase awareness of gender disparities and to inform future initiatives for improvement in care, aiming to identify differences in patient characteristics, delays to care, treatment strategies, and outcomes of gender.	High
15	Tsang et al. [21]	2011	Systematic Review	30%	325	A systematic review of landmark cardiovascular clinical trials to evaluate enrolment rates of women and to determine the association between age and enrolment.	Low to Moderate
16	van Oosterhout et al. [22]	2020	Systematic Review & Meta-Analysis	40%	27	Review of the extent of sex differences in symptom presentation in patients with confirmed acute coronary syndrome (ACS).	High
17	Worrall-Carter et al. [23]	2017	Systematic Review & Meta-Analysis	23-40% Average 32%	25	The review aimed to analyse studies that report risk stratification of non-ST elevated myocardial infarction (NSTEMI) patients by gender to test the hypothesis that women are less likely to be risk stratified as high risk in comparison to men.	Low

On review of the included papers, they can be loosely summarised by focus/aims into the following categories: four articles focused on risk factors and highlighted subgroups, two focused on presentation, nine papers focused on treatment, and two focused on current research. In view of the varying aims of each study, a comparison of results is not applicable, however, a few key findings of numerical value are highlighted here.

Nguyen et al [16]. studied the reasons for pre-hospital delays in presentation with myocardial infarctions (MI); their study focused on age and sex differences and found that women were 10-18% more likely to present later than men following the onset of symptoms. However, it is important to highlight the multiple limitations highlighted within this study. The authors noted significant selection bias with limited generalisability due to small sample sizes, resulting in the underrepresentation of women, accounting for 20-50% of participants within the included studies [16]. Further research is needed to enable action to explore and address delays noted in the presentation, particularly for vulnerable groups.

Shah et al. [20] explored gender disparities in in-hospital care and outcomes in patients following ST-elevated myocardial infarction (STEMI). This study deemed there to be worse outcomes for women, with an unadjusted rate of in-hospital mortality being higher in females (p<0.00001 statistically significant, I2 = 58% “moderate heterogeneity”), which have seen little change over the past 20 years. However, once these results were adjusted for age alone, females no longer had a significantly increased mortality rate (p<0.0001 statistically significant). It is however worth noting that women were underrepresented within this study, accounting for 30% of participants. Shah et al. concluded that age remains the most significant contributor to gender disparity in mortality and they attributed this to the tendency that women present 5-10 years later than men due to premenopausal estrogen levels delaying the development of CVD [20]. However, it should be acknowledged that comorbidities, suboptimal treatment including drug therapy, and delays in care are also known to be contributing factors to excessive post-event female mortality rates.

Colella et al. examined rates of referral for outpatient cardiac rehabilitation and reported a sex bias, with women (39.6%) significantly less likely to be referred when compared to men (49.4%; odds ratio 0.68, 95% confidence interval 0.62-0.74); this sex bias was deemed by the authors as statistically significant, however, the level of heterogeneity was also considered significant (I2=90%) and therefore further research is recommended [10].

Giorgini et al. [11] studied clinical outcomes following anti-hypertensive treatment; their study findings diverged from their hypothesis - that anti-hypertensive treatment may cause poorer outcomes in women. Their meta-analysis reached statistical significance demonstrating sex differences in outcomes, with men at greater risk of CV events when treated with the same anti-hypertensive therapy (odds ratio 1.25, 95% confidence interval: 1.17, 1.33, p <0.001; I2: 40.17%). However, there were multiple limitations highlighted in this study, including questionable levels of heterogeneity due to differing trial types, drug types, and dosages and an underrepresentation of women at 33.5% of included participants [11]. Further research is recommended to investigate likely gender differences in hypertension to clarify if a gender-orientated approach to treatment is desirable.

Although multiple limitations are acknowledged within all the included articles, the results are irrefutable regarding the significant level of gender-based inequality in CV health despite the high level of underrepresentation of women throughout each reviewed article. The need for further research is clear. The reasoning for such disparities is discussed in depth in the next section.

Discussion

Reoccurring themes were noted throughout the included studies, with 13 selected for investigation. In Table 3, these topics have been used as subheadings to form a structure for discussion within this umbrella review.

**Table 3:** Common Themes / Summary of Findings Key of Articles: 1 = Backholer et al. [8], 2 = Caceres et al. [9], 3 = Colella et al. [10], 4 = Giorgini et al. [11], 5 = Hamill and Ingram [12], 6 = Lewey et al. [13], 7 = Mannem et al. [14], 8 = Meads et al. [15], 9 = Nguyen et al. [16], 10 = Oertelt-Prigione et al. [17], 11 = Pancholy et al. [18], 12 = Panza et al. [19], 13 = Shah et al. [20], 14 = Tsang et al. [21], 15 = van Oosterhout et al. [22], 16 = Worrall-Carter et al. [23], 17 = Zhao et al. [4].

Common Themes / Summary of Findings			
Reoccurring Themes		Citing Articles	
Need to Focus on Gender-Specific Prevention Resources		1, 2, 3, 5, 6, 9, 11, 16, 17	
Gender Disparity and Education / Socioeconomic Status		1, 6, 9, 12	
Delay in Presentation		1, 5, 6, 8, 9, 13, 15, 17	
Gender Differences in Presentation		1, 3, 4, 5, 6, 9, 10, 11, 13, 15, 16, 17	
Poor Assessment / Misdiagnosis		5, 11, 13, 15, 17	
Delayed onset of Symptoms / Age / Post Menopause		4, 5, 9, 13, 14, 15, 16, 17	
Gender Inequalities in Accessing Treatment		1, 3, 4, 5, 6, 7, 9, 11, 13, 16, 17	
Gender Inequalities in Treatment		3, 4, 5, 6, 11, 13, 14, 16, 17	
Gender Disparity in Secondary Prevention		3, 6, 8, 11, 16, 17	
Underrepresentation of Women in Research		3, 5, 7, 9, 10, 11, 13, 14, 15, 16, 17	
Need for Further Research into Gender Differences / Inequalities		1, 2, 4, 3, 5, 6, 7, 8, 9, 10, 11, 12, 13, 14, 15, 16, 17	
Need for Further Understanding of Causes of Sex Differences		1, 4, 5, 10, 11, 13, 14, 15, 17	
Need for Gender-Specific Clinical Guidance		1, 2, 3, 4, 5, 6, 9, 10, 13, 14, 15, 16	

For instance, a paper by Mehta et al. highlights a section of the American Heart Association’s (AHA) scientific statement that states that sex differences in clinical presentation are to the detriment of women, leading to delayed identification of symptoms, assessment, diagnosis, and management [24]. Through this review, the investigator explores the reason for this ongoing inequality in CVD. As the common themes are investigated, it has been noted that the implications and outcomes of these themes frequently overlap.

Need to Focus on Gender-Specific Prevention Resources

Although in recent years there has been more of a focus on gender disparities in medicine, there remains a reduced awareness of CVD risk and the implications this poses to women. The need for more female-specific resources to aid in increasing awareness and educating both the general population and healthcare professionals alike has been highlighted by over half of the included articles. Backholer et al. [8] recommend that the focus should be on intense risk detection, particularly among disadvantaged women through targeted prevention and management strategies. They suggest that to meet the needs of socially disadvantaged women, resources should be aimed at more non-traditional risk factors to meet their needs [8]. This is echoed by Caceres et al. [9] who highlight the plight of sexual minorities - an often-forgotten subgroup. Understanding the role of gender on differing lifestyles and cultures is required to enable allocations of often limited preventative resources [8].

Gender Disparity and Education / Socioeconomic Status

Socioeconomic status remains a strong independent risk factor for all CVD outcomes with disproportionately stronger effects on women [8]. Similarly, Panza et al. [19] note that gender remains one of the most reported forms of discrimination particularly, against women. The need to examine vulnerability to CVD amongst a diverse sample within stigmatized groups remains evident. A paper by Backholer et al. [8] is one of many that report on sex differences within CVD being influenced by socioeconomic status and education. They studied the impact and implications of this and recommend that to reduce such inequalities, prevention and treatment strategies should be tailored to gender within local contexts; this includes the continued development and implementation of sex-specific CVD risk scores within global practice and clinical guidance to enable assessment of socioeconomic disadvantage [8].

Delay in Presentation

Delay in presentation is widely reported as a gender disparity leading to poorer outcomes amongst women. Nguyen et al. [16] have studied the reasons for pre-hospital delay of presentation with an MI. Their study focused on age and sex differences. Interestingly low education and having a partner with low education contribute to the presentation delays seen amongst men, along with not asking for help, declining ambulance transport, experiencing early musculoskeletal pain, developing a non-Q wave MI, lack of consistency between expected and experienced symptoms and lack of awareness of treatment. However, a history of previous MI was associated with a reduced delay in presentation. Differing factors were attributed by Nguyen et al. to delays seen in the presentation of women, with women being older and single, with a history of previous MI and frequently being alone at the time of onset of symptoms; they also did not want to trouble anyone, were more likely to delay seeking medical attention, particularly in comparison to women who did not have such characteristics. From clinical experience following discussions with patients’ post-CVD events, these proven factors associated with delays in presentation are commonly heard in clinics [16].

Nguyen et al. summarised their findings stating that the median duration of pre-hospital delay in women ranged from 1.8 to 7.2 hours whereas the time range for men was smaller 1.4 to 3.5 hours. Patients arriving at the hospital within two hours of the onset of symptoms are more likely to be male, with women more likely to have a later presentation. Women were 10% more likely than men to present after two hours after the onset of symptoms; this gap widened to 18% for a presenting cut point at six hours [16].

Another key point raised amongst the reviewed studies was the impact of misinformation regarding the belief that women suffer and present with CVD the same as men. Hamill and Ingram argue that this belief results in ineffective recognition of cardiac signs and symptoms amongst women and healthcare professionals, leading to delays in seeking help, misdiagnosis, and referral for treatment [12].

Gender Differences in Presentation

**Figure 2:**
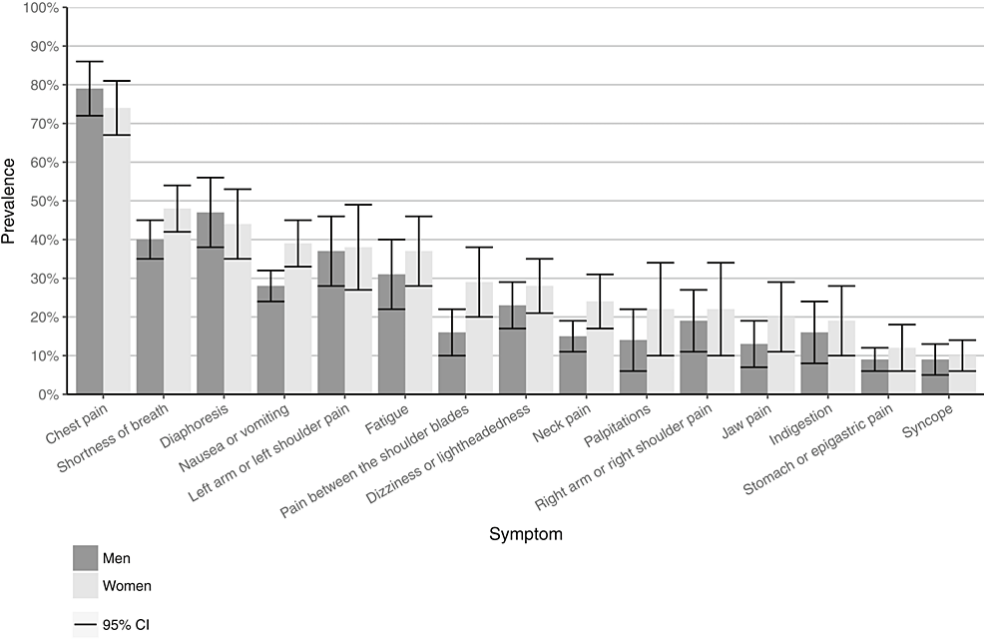
Results of the meta‐analysis of the pooled prevalence and corresponding 95% CI for all symptoms for ACS in women and men. Acute coronary syndrome (ACS); permission obtained from authors to reproduce figure 2 [22]

As previously mentioned within this review traditionally men are used as the standard frame of reference for all diseases shared by all genders, Hamill and Ingram astutely refer to this as the “male template” [12]. Three-quarters of the articles reviewed highlighted gender differences in presentation as a major factor leading to gender-based inequalities in CV health.

van Oosterhout et al. [22] focus on sex differences in symptom presentation in acute coronary syndromes (ACS); they state that despite substantial overlap there are many sex differences in presenting symptoms. They report that women have higher odds of experiencing pain between the shoulder blades, nausea/vomiting, and shortness of breath, and have lower rates of presenting with chest pain or diaphoresis. Women had lower odds of presenting with chest pain in comparison to men [22]. This is clearly demonstrated in Figure 2 below showing the pooled prevalence of symptoms of ACS symptoms taken from the meta-analysis.

These findings are echoed by Hamill and Ingram [12] who recommend that clinicians should be educated to recognise that women are more likely to present with generalised chest discomfort/pain, fatigue, syncope, nausea/vomiting, back pain, palpitations, shortness of breath and sense of dread. Yet men typically present with chest pain and diaphoresis, which are often the symptoms of MI commonly characterised in the media [12]. This contributes to the notion of CVD being a man’s disease and leads to the misconceptions of signs and symptoms to monitor for, resulting in a lack of awareness/recognition of symptoms and therefore ultimately a delay in presentation.

Van Oosterhout et al. argue that symptoms of ACS should no longer be labelled with “typical” and “atypical” symptoms for both sexes, stating that instead there should be clinical recognition of sex differences proportionate to the many symptoms that overlap [22]. Such terms have become outdated given that sex differences in the presentation have been widely recognised and reported since early 2000.

Clinical research into differences in symptoms and diagnosis is underrepresented, despite the differing presentations across sexes being well known, and ultimately being widely blamed for the delay in treatment of women having an MI [17]. This is reflected within this umbrella review with only two of the included papers being focused on presentation.

Poor Assessment / Misdiagnosis

Males are more likely to believe their symptoms are cardiac in nature and are seen to be encouraged by bystanders to seek medical attention while females often attribute their symptoms to anxiety. Shah et al. advise in view of this, and given that females tend to be less reluctant to use medical services, then greater awareness is needed for both public and healthcare professionals alike to understand presentations and reduce possible delays in care [20].

There is also evidence suggested by Pancholy et al. that women have a higher prevalence of prehospital misdiagnosis of ST-elevated myocardial infarction (STEMI) [18]. Hamill and Ingram [12] cite a cohort study by Golden et al. (2013) whose study of 206 patients showed that women were less likely to be advised their symptoms could result in heart disease and were more likely to be diagnosed as gastric reflux (p=0.03 statistically significant) or “unknown aetiology”, despite similarities in a presentation seen in both genders and ACS diagnoses having been considered in all the patients included within the study [12].

These findings are not surprising. Through clinical experience, the investigator recalls many consultations with female patients’ post experiencing CVD events who remain angry that they had experienced a delay in diagnosis and treatment, feeling bereft having suffered with their symptoms for longer or being made to feel like a “fraud” or “nuisance” following multiple presentations in primary and secondary care without resolution.

van Oosterhout et al. [22] lead their recommendations with the need for improved recognition of symptoms and sex differences amongst medical professionals to ensure prompt assessment and treatment. It is widely recognised that the symptoms of ACS that women more commonly present with are easily attributed to other causes and therefore lead to a delay in diagnosis or misdiagnosis. Therefore, van Oosterhout et al., recommend the need for validation of diagnostic tools which consider sex differences at presentation [22].

Incidence of CVD in women is about a third of that seen in men during middle age, with CVD occurring in men a decade earlier than women, therefore Zhao et al. suggest this may result in a misconception that CVD is less common in women and therefore does not require as an intensive preventative therapy as men [4].

Delayed Onset of Symptoms / Age / Post Menopause

Multiple studies highlight that females consistently have a higher prevalence of comorbidities at the time of CV events in comparison to men. Older age, diabetes, hypertension, and dyslipidemia are commonly associated with women, while men are more likely to smoke [18].

Age is a major contributing factor to gender inequalities seen within CV health and has been repeatedly acknowledged by many of the included articles within this review. Nguyen et al. highlight that women are older at the time of their first CV event which directly correlates with a significant delay seen in seeking medical attention, often resulting in poorer outcomes [16].

Shah et al. undertook a meta-analysis of gender disparities in hospital care and outcomes in patients following a STEMI, which showed that within the pooled baseline characteristics, women were older than men at the time of their STEMI with an average age of 70.2 ± 3.1 years, in comparison to men with an average age 61.1 ± 2.2 years [20]. Women were also more likely to present with diabetes, hypertension, a history of prior stroke, and cardiogenic shock at the time of presentation, whereas men were significantly more likely to be smokers or have a history of prior MI. Shah et al. conclude that age remains the most significant contributor to gender disparity in mortality and they attribute this to the tendency that women present 5-10 years later than men due to premenopausal estrogen levels delaying the development of CVD [20]. However, it should be acknowledged that comorbidities, suboptimal treatment including drug therapy, and delays in care are also known to be contributing factors to excessive post-event female mortality rates.

It is prudent to note however as with all the studies examined within this umbrella review there is an underrepresentation of women with 31% of participants being female leading to a lack of data for younger females (<60 years). Therefore, further investigation is needed into the true extent of the gender gap across all age groups [20].

Please refer to the Risk Factor section in this article for further investigation into the gender differences contributing to delays in the onset of symptoms.

Gender Inequalities in Accessing Treatment / Gender Inequalities in Treatment

Pancholy et al. state that healthcare utilization by women is frequently found to be suboptimal in comparison to men, particularly those presenting following STEMI. They observed that during the thrombolysis era, women were less likely to receive thrombolysis, and despite reperfusion therapy correlating to higher survival rates in women than men, women remain less likely to receive primary reperfusion therapy [18]. Pancholy et al. suggest that women are in poorer conditions at the time of presentation due to delays and are therefore overlooked for possible newer evidence-based therapies [18]. Zhao et al. echo these findings, stating that studies show women are likely to experience a significant delay in receiving medical treatment to reduce the risk of incidents or recurrent CV events [4]. These disparities can be seen within subgroups with combined gender and racial inequalities. Lewey et al. report that in comparison to White men, White women are approximately 10% less likely and Black women 25% less likely to undergo cardiac catheterization following MI [13].

Gender differences demonstrated by the lower rates of angiography in women are attributed in some studies to the use of secondary prevention, while others advise that an “androcentric gender bias” in the diagnosis and treatment of CVD should actually be considered as discrimination. The WISE (Women’s Ischaemia Syndrome Evaluation) Study cited by Worrall-Carter et al. [23] suggests that biological differences in the cause and effects of CVD could be the essence of gender bias in the treatment of ACS. Worrall-Carter et al. acknowledge a treatment risk paradox revealed through their study that patients at higher risk of non-ST elevated myocardial infarction (NSTEMI) were in fact less likely to receive the most effective invasive cardiac procedures [23].

The underrepresentation of females highlighted by Mannem et al. in their study into sex differences and outcomes following percutaneous intervention in patients with chronic total occlusion, despite the proven effectiveness of the procedure in both males and females, suggests a possible selection bias by referring clinicians, with females less frequently referred for invasive management of atypical symptoms [14].

Gender Disparity in Secondary Prevention

As previously mentioned, misconceptions of women being at lower CVD risk can result in reduced prioritization of CV prevention amongst both women and clinicians [13]. Similarly, patients with known CVD can remain under both primary and secondary care simultaneously resulting in a lack of clear case management and often resulting in delays or lack of up-titration of cardioprotective therapies. Zhao et al. report a lower prevalence of prescribed aspirin, statins, and angiotensin-converting enzyme inhibitors (ACEi) amongst women with an established risk of CVD, whilst a lower prevalence of prescribed diuretics was noted in men [4]. Lewey et al. also suggest that with a larger proportion of patients requiring medical management for both primary and secondary prevention of a CV event in comparison to invasive CV procedures, then efforts should be made to reduce non-adherence to medical management to improve gender disparities as opposed to addressing other evidence-based therapies [13].

It has been proven that gender bias is clear throughout the spectrum of CV health, with cardiac rehabilitation (CR) not being an exception despite the introduction of systematic referral strategies. Colella et al. reported through their meta-analysis (including data from the past decade) that referrals to outpatient cardiac rehabilitation programs remain sub-optimal, with an average rate of referral for women standing at 39.6% [10]. Colella et al. were able to reveal that men were 1.5x more likely to be referred to CR programs than their female counterparts; this is despite CR being recommended as a standard for secondary prevention for both men and women [10]. Colella et al., conclude that “female patients deserve the best available cardiac care, and this involves the eradication of any bias for referral to outpatient CR” [10]. This is a sentiment the investigator fully endorses as a cardiac rehab nurse specialist who is sadly only too aware of this inequality from their many years of clinical practice.

Although not gender specific, weight stigma within healthcare is recognised by Panza et al., who state that patients with obesity are less likely to undergo health screenings, more likely to delay presenting or even avoid seeking help; therefore increasing the likelihood of CVD remaining undiagnosed or poorly managed through lack of treatment [19]. A solution to this is also suggested by Panza et al., who recommend that healthcare professionals could benefit from training regarding discrimination and its implications for their patient’s health. Focus on reducing stigma in health can improve the patient experience of more vulnerable groups [19].

Underrepresentation of Women in Research, the Need for Further Understanding of Causes of Sex Differences and the Need for Further Research Into Gender Differences / Inequalities

The underrepresentation of women within research was the single theme that was unanimously advocated by all the included studies within this umbrella review. There is therefore no denying the need for an increase in the inclusion of women.

Tsang et al. evaluated the prevalence of women’s enrolment in landmark randomised CV trials. Their study showed an increase of 5% over 12 years in the enrolment rate of women in CV trials, from 27% in 1997 to 32% in 2009. The average female representation within the studies included in this umbrella review was 33.9%, which correlates with these findings [21]. Tsang et al. reviewed the reasons for such underrepresentation and were able to prove a correlation between enrolment rates of women and age at recruitment [21]. Trials that included older patients focusing on coronary artery disease, heart failure, and arrhythmia were associated with increased female participation. Tsang et al. were also able to adjust their findings to account for age and gender-specific differences seen in population data showing disease prevalence, this then suggested there was only a 3-13% lower than expected rate of recruitment to trials. They concluded that persistent low enrolment rates of women in CV clinical trials are mostly driven by an epiphenomenon of age-recruitment bias leading to enrolment to be drawn from cohorts of patients with CVD who are predominantly male. Therefore Tsang et al., recommend that strategies should be made to recruit the older population in clinical trials to enable a more accurate representation of women in CV clinical trials [21].

Piepoli et al. [2] highlight those women who were young, older and from ethnic minorities remain under-represented in clinical trials. They further note that there is currently a gap in evidence regarding whether there is an associated increased risk of CVD with female-specific conditions or if this occurs independently from the most common CVD risk factors [2]. Similarly, whether the inclusion of female-specific conditions could improve risk classification remains to be investigated. The European Society of Cardiology (ESC) (2019) guidelines also highlight that very few randomised trials of statin therapy are reported to be of significant benefit to women regarding CV risk. However, ESC acknowledges this is mostly due to women being under-represented within statin trials [25].

It is thought that women have less belief than men in the safety and effectiveness of CV medicine, with poorer adherence also noted [4]. Therefore, increasing the participation of women within clinical trials would not only improve current evidence-based practice, enabling more clinicians to better advocate treatment but also provide women with the reassurance that the treatments are proven to be safe and effective with the ultimate aim to improve adherence to prescribed drug therapy.

Giorgini et al. whose study examined differing clinical outcomes between genders with anti-hypertensive therapy, also concluded that further research is needed to clarify if a more gender-orientated approach to anti-hypertensive treatment is needed [11]. Zhao et al. advocate the need for future research “to determine underlying causes of observed sex differences and to develop tailored strategies to optimize the use of evidence-based medication for both women and men” [4].

Meads et al. [15]; Caceres et al. [9] and Shah et al. [20] all make recommendations for further research into causes of health inequalities and gender differences along with the need to utilise medical records and registries to ensure data is kept updated to include better representation of subgroups such as sexual minority groups.

As already discussed, sex differences in symptoms for patients with ACS have been widely acknowledged since the early 2000s; however, these factors have hardly been reviewed since. van Oosterhout et al. note that in the past 20 years, the focus of research has been on sex differences in the pathophysiology, symptom presentation, and outcomes of ischaemic heart disease, however, many gaps remain in the understanding of mechanisms for symptoms and pathophysiology in ACS in women and therefore further research is needed [22].

Need for Gender-Specific Clinical Guidance

Due to the underrepresentation of women within CV clinical trials, there is reservation from experts to generalize research findings into clinical practice, which leads to suboptimal care of women within CV health [21]. One of the key pillars of healthcare is the reliance on evidence-based medicine, however, with a lack of gender-specific evidence clinicians cannot be confident that they are acting in their patients’ best interests. There is an undeniable inequality in treatment which is summarised by Hamill and Ingram who states that “until guidelines reflect the need for more gender-specific care, recognition and implementation by the majority of the healthcare profession will not happen” [12]. Current guidance is discussed in greater detail prior to the conclusion of this review.

Risk factors

Increased age, hypertension, diabetes, and dyslipidemia are reoccurring risk factors for CVD reported within the included articles [4, 8-23]. Shah et al. advise that gender disparities in in-hospital outcomes can largely be explained by differences in age. However, they report that comorbidities, delays in care, and sub-optimal treatment also contribute to and need great improvement at a global level [20]. The meta-analysis undertaken by van Oosterhout et al. reported that women presenting with ACS were more likely to be older, and more frequently present with comorbidities such as diabetes and hypertension [22]. Both studies recognised that the male participants were more frequently smokers.

Gaggin and Oseran write that although in the United States (US) CVD is the leading cause of death in both genders; the awareness of CVD in women is very poor resulting in misdiagnosis or delay in diagnosis of MI. Education is required to emphasize that post-menopausal women have a dramatic increase in risk of CVD, which equates to one in three deaths [26].

A study by Hyun et al. focused on the utilization of risk factor assessment and management of CVD within primary care (The TORPEDO trial). The study reviewed data from 53,085 patients (58% female) and found a higher incidence of missing or omitted risk factor data within the female records (such as the absence of smoking or diabetic status) leading to the inability to accurately score an absolute risk of CVD [27]. It is also worth noting that women with diabetes have a 40% greater risk of coronary heart disease (CHD) and a 30% increased risk of stroke than men. Hyun et al. conclude that younger women are particularly penalised through the denial of preventative therapy [27].

It is clear within the included studies that further research is required to understand the sex differences seen in both presentation and symptoms of CVD. A brief overview of the current understanding/ reasoning regarding gender-specific risk factors is provided below.

Age/ Menopause

As already discussed, age has been highlighted as a major factor contributing to increased CVD risk in women. Pre-menopausal women are seen to have a lower incidence of CV events, however, after menopause incident rates rise steeply and parallel with those seen in men. It is thought that estrogen has cardioprotective properties and so as estrogen levels decrease with ageing and particularly after menopause, this level of protection subsides. In some studies, estrogen has shown possible beneficial antioxidant and antiplatelet actions which improve endothelium-dependent vasodilation [28, 29].

In a review of current literature, Mehta et al. state that the understanding behind the mechanisms of estrogen on CV health remains underexplored, however, the effects of estrogen on the vascular system are recognised as increasing nitric oxide leading to vasodilation, regulation of prostaglandin production, and inhibition of smooth muscle proliferation [24]. Studies also show that with the reduction in estrogen at menopause there is an increase in endothelial dysfunction and lipid deposition precipitating atherosclerosis over time [24]. Yet despite the cardioprotective effects of estrogen, the use of hormone replacement therapy (HRT) is not recommended for primary prevention of CVD in postmenopausal women. Similarly, women who are already prescribed HRT are advised to cease following CV events [24].

Lipid Profiles

Premenopause estrogen levels raise high-density lipoprotein (HDL) and lower low-density lipoprotein (LDL) levels [28]. Durrington [30] recommends that serum lipid levels vary during the phases of the menstrual cycle, with cholesterol and triglycerides building up to a peak at mid-cycle at the time of ovulation and declining during the subsequent progestogenic phase. Durrington attributes the rise in serum cholesterol predominantly due to an increase in HDL cholesterol [30]. Post menopause, however, there are contradictory reports within studies as to which lipoproteins are responsible for the abrupt rise seen in serum cholesterol levels in women in their late 40s and early 50s. Durrington surmises that it is thought to be due to a significant rise in LDL however there are also gradual rises noted in very low-density lipoprotein (VLDL) and HDL at the same time [30].

*Hypertension* 

Hypertension is a greater risk to females, yet the pathophysiology of hypertension and the age-dependent incidence of hypertension in women and men remains poorly understood. Dorobantu et al. state that hormones are thought to be a prevalent factor behind age and gender disparity in hypertension [31]. Premenopause estrogen suppresses the angiotensin receptor type 1 and stimulates the angiotensin receptor type 2, however, postmenopause the activation of the renin-angiotensin system is thought to play a role in increasing hypertension in women [31].

As previously discussed, the Giorgini et al. study diverged from their hypothesis, with their findings showing that men are at greater risk of CV events when treated with the same anti-hypertensive therapy [11]. However, in view of some gender differences noted in CV morbidity and mortality, further research is recommended to determine if the suspected gender differences in hypertension require the development of a gender-orientated approach to anti-hypertensive treatment.

Diabetes

Diabetes was highlighted by Hamill and Ingram as a significant confounding factor in the incidence of CVD events in women, however, they report that further research is needed into possible reasoning for this [12].

On investigation, the incidence of coronary heart disease in diabetic women was 44% greater than that seen in men with diabetes [32]. Peters et al. suggest that there is likely a greater deterioration in CV risk profile along with prolonged exposure to adverse levels of CV risk factors seen among prediabetic women in comparison to their male equivalent, it is thought that greater levels of adiposity are responsible for the excess risk of diabetes related to CVD in women [32].

Clemens et al. also acknowledge that women with type 1 and type 2 diabetes are known to have a higher risk of CVD, stroke, heart failure, and mortality when compared to men [33]. Common baseline sex differences observed by Clemens et al. were heart failure, chronic kidney disease, and stroke. Interestingly they also noted that women with hypertension and diabetes were considered to be at higher risk of heart failure and more commonly with preserved ejection fraction. They note these sex differences observed could be attributed to body composition, hormones, endothelial inflammation, and microvascular function [33].

A key point both Clemens et al. [33] and Peters et al. [32] agree on is that further research is required into this subgroup.

Smoking

Although this umbrella review has acknowledged that smoking as a risk factor has been more commonly associated with men, the INTERHEART study cited by Mehta et al., also found that a history of smoking had a stronger association with MI in men, although current smoking status did not appear to show significant variation in sex [24].

However, smoking and obesity appear to have a higher prevalence amongst younger women with known MI in comparison to older women. Mehta et al. hail smoking as the “single most important preventable cause of MI in Women and a leading cause of MI in women <55 years of age, increasing their risk 7-fold” [24].

However, this risk can be significantly reduced following 1 year of smoking cessation [34]. Therefore, these findings support the argument for improving health education amongst women and support the call for gender-specific prevention resources.

Polycystic Ovary Syndrome (PCOS)

Although many of the studies reviewed have alluded to female-specific conditions being linked to increased risk of CVD, very few are able to provide any scientific evidence for this, hence the calls for further research into CV health and female-specific conditions. On investigation Dorobantu et al. associate an increased prevalence of hypertension and CHD to PCOS [31]. PCOS is an endocrine condition that affects 10% of females of reproductive age; it is associated with anovulation, hyperandrogenism, increased body mass index, central obesity, insulin resistance, and hypertension. Dorobantu et al. report there is mounting evidence linking PCOS to diabetes, hypertension, and CHD, with glucose intolerance, diabetes and hypertension already being known risk factors of CVD and most commonly prevalent in women [31]. Interestingly Dorobantu et al. also highlight the presence of more significant obstructive coronary artery disease and multivessel disease in women with PCOS on angiography when compared to women without, the reasoning for this remains disputed, however it is thought it could be due to impaired endothelial function [31]. The WISE study cited by Dorobantu et al. reported that postmenopausal women with clinical features of PCOS have a 10% lower five-year survival rate for CHD in comparison to healthy women [31].

Emerging presentations

Gender differences seen in the presentation have been widely acknowledged throughout the included studies within this umbrella review. Although as noted by van Oosterhout et al. understanding of sex differences within CV health has progressed, there remain multiple gaps in the understanding of the mechanisms for such sex differences in symptoms and indeed pathophysiology of ACS in women [22].

The van Oosterhout et al. (2020) study cites the American Heart Association’s definition of ACS as either NSTEMI, STEMI, or unstable angina, with the guidance that ACS is diagnosed through the presence of symptoms of MI, new ECG changes, and elevated cardiac enzyme. They note that younger women more commonly present with coronary artery spasms and vascular dysfunction (type II ACS), whereas younger men more frequently present with coronary artery obstruction (type I ACS). At all ages women with ACS more frequently present with plaque erosions rather than ruptures in comparison to men [22].

Mehta et al. state that gender differences in the clinical presentation have “consequences for timely identification of ischemic symptoms, appropriate triage, and judicious diagnostic testing and management” [24]. van Oosterhout et al. also note that symptoms women present with are highly common in other conditions, complicating diagnosis [22]. Misdiagnosis, delayed treatment/revascularisation, and potential higher mortality rates were points echoed by many experts included in this review when discussing sex differences and CV health.

Many experts argue that the aetiology of CVD in women is complex with obstructive disease and microvascular disease playing differing roles [12]. Mehta et al. recognize that the underlying causes for pathophysiological sex differences seen in MI are multifactorial, with biological sex characteristics in women being attributed to differences seen in plaque characteristics such as rupture vs erosion and the prevalence of coronary artery spasm and SCAD [24].

Plaque rupture is thought to be the most common cause of fatal MI as discovered through studies in autopsy, being responsible for 76% of male and 55% of female fatal MI [24]. However as reported by Mehta et al., plaque erosion has a far greater prevalence in younger women and has also been characterised in patients with STEMI and NSTEMI following thrombus extraction. Plaque erosion is reported to account for 27% of patients with STEMI and 31% of NSTEMI, with female sex and premenopausal status being the only two risk factors highlighted to enable prediction of this type of thrombotic coronary lesion following studies conducted at autopsy [24]. It is however prudent to highlight that Mehta et al., acknowledge a limitation to their study in the use of an age-matching algorithm, which in turn causes a lack of representation of women within the study particularly those of a younger age known to have a higher prevalence of plaque erosion. Plaque rupture is deemed to be particularly rare in premenopausal women, providing further support for the protective effect of estrogen [24].

In support of gender differences seen in CV health, Stoberock et al.'s study of carotid stenosis also found sex differences in both presentation and treatment outcomes [35]. However, they also concluded the reason for such differences across genders remains unclear, but suggest it could be attributed to “biological, anatomical (smaller vessel diameter) or hormonal reasons as well as a protracted development of atherosclerotic changes in women” - attributable to age offsetting symptoms seen premenopause [35].

It is worth noting that due to sex differences seen at the time of presentation, Mannem et al. acknowledge the need for consideration of gender-specific treatment strategies [14]. Their study of sex differences and outcomes of interventions for chronic total occlusions (CTO) highlighted that although favourable success rates were seen in CTO due to lesions frequently being shorter with blunt stumps and good collateral supply, women often experienced more complications post-procedure. These complications include coronary perforation, contrast-induced neuropathy, radiation exposure, and bleeding. Mannem et al., advise this also translates to higher mortality rates seen post coronary artery bypass (CABG), and more difficulties noted in recovery in women [14].

On commencing this project, the investigator hoped this umbrella review would lead to further understanding of emerging female dominant cardiac conditions such as takotsubo, myocardial infarction in the absence of obstructive coronary artery disease (MINOCA), and SCAD, however, surprisingly these conditions were not mentioned within the SR and MA studies included within this review. This may be partially due to these conditions still being researched, SCAD research particularly remains in the early stages.

Takotsubo Cardiomyopathy

Takotsubo is a type of cardiomyopathy that is not thought to be genetic and has a higher prevalence in women than men, it is most common in women over 50 years (likely postmenopause) and is thought to affect 2500 people in the UK each year [36].

Takotsubo was first described in Japan in 1990 and named after a Japanese octopus trap thought to be similar to the shape of the left ventricle apical ballooning associated with this condition. This condition is often referred to as “broken-heart syndrome” and although the exact cause remains unknown it is thought to occur due to extreme stressful emotions or a physical event such as illness or pain. Experts believe these events cause an excess of hormones such as adrenaline to be released which weakens the cardiac muscle. However, 30% of cases have no obvious trigger [36, 37].

Takotsubo is transient and often improves with treatment. However, recent research suggests the condition could have a more prolonged effect on cardiac function, and therefore ongoing cardiology follow-up is recommended, for some the condition may reoccur if they suffer from a similar stressor in the future [36]. Kuo et al. conducted a case series that was inconclusive due to its limitations associated with being a single-centre study including a small pool of participants, however, they make recommendations for further research into the impact of reduced levels of estrogen seen in postmenopausal women potentially predisposing them to takotsubo [37].

Myocardial Infarction in the Absence of Obstructive Coronary Artery Disease (MINOCA)

As already discussed, plaque rupture remains the leading cause of MI, however following analysis of 27 large clinical trials and registries, Vogel et al. report 6-15% of patients with MI had no evidence of obstructive coronary artery disease [1].

Vogel et al. state that MINOCA is more prevalent in women (10.5%) than men (3.4%) (p<0.0001) [1]. MINOCA, a term used to describe conditions caused by coronary mechanisms such as coronary artery dissection, coronary spasm, and coronary emboli, should only be considered in patients with a definitive diagnosis of MI, with a non-obstructive disease on angiogram, and no other underlying clinical factors leading to myocardial injury without ischemia. Therefore, it is recommended that cardiac magnetic resonance imaging (MRI) be used to exclude myocarditis, takotsubo, and other cardiomyopathies [1]. It is also worth noting that 25% of patients with MINOCA have ongoing symptoms of angina [1].

Spontaneous Coronary Artery Dissection (SCAD)

SCAD is a rare cardiac condition that as yet cannot be predicted or prevented. Research into this condition remains in the initial stages, with causes also yet to be determined, however, some common associating factors have been established: pregnancy and post-partum; menopause; fibromuscular dysplasia; connective tissue disorders, extreme stress, and extreme exercise or emotional stress [38].

Mehta et al. recommend that SCAD should be suspected in any young woman presenting with an ACS without typical atherosclerotic risk factors [24]. SCAD has been reported across a wide age range of 18-84 years; however, the majority of cases are in young to middle-aged women. Current data supplied by Beat SCAD UK (2020) shows that SCAD affects 90% of women and 10% of men, with most aged 44-53 years. 10% of SCAD events occur during or soon after pregnancy, with 50% of all postpartum coronary events attributable to SCAD. Exercise is reported as the most common trigger of SCAD seen in men [38].

There are currently no definitive guidelines for optimal treatment strategies for patients with SCAD. Vogel et al. stipulate in their report that further urgent research is needed to “address the uncertainties about prevalence and treatment of SCAD as well as post-SCAD lifestyle modifications and medical therapies” [1]. Vogel et al., also hail the use of the EURObservational Research Programme and other SCAD registries [1].

In the clinic, the investigator has noted an increase in the number of patients presenting with SCAD, and their service has contacted the lead research centre based in Leicester. It is important to recognize the role that an individualised cardiac rehabilitation program can play in aiding recovery and increasing confidence post-SCAD event, along with onward support with recommended lifestyle modifications.

Current guidance

The National Institute for Health and Care Excellence (NICE) Clinical Guidance 181 [39], provides no variation for women in terms of prevention, diagnosis, or treatment of CVD, similarly ESC (2019) guidance provides minimal support to clinicians in this area [25]. Interestingly, the latest NICE impact report for CVD prevention [40] highlights a need to focus on reducing inequalities in detection, management, and outcomes, however, the report makes no reference to gender disparities or sex differences.

Hamill and Ingram highlight a disparity in current guidelines resulting in inequality in treatment across genders [12]. They cite NICE guidance for investigation and diagnosis of recent onset chest pain [41]; this guidance currently advises clinicians that symptoms should not be assessed or defined by gender, and sex differences should not be accounted for. On investigation, Hamill and Ingram [12] report that by following this guidance a female attending with non-anginal chest pain or atypical symptoms, regardless of risk category, should not be offered coronary angiography. However, in comparison if a male (any age) with atypical symptoms is in the high-risk category or a male of low risk with atypical symptoms aged over 60 years, then a coronary angiogram should be offered. Hamill and Ingram elaborate further stating that females only receive equal treatment at the point of presenting with an acute MI with the criteria based upon blood and electrocardiogram (ECG) changes. They conclude that until guidance is changed to enable gender-specific care then the delivery of healthcare will remain unequal [12].

The investigator agrees with this assessment; healthcare is managed by set strategies and clinical guidelines, until these change and clinicians are educated in recognizing gender differences and gender-specific treatments are implemented led through evidence-based medicine, then the unequivocal gender-based CV health inequalities highlighted throughout this umbrella review will sadly remain unaddressed to the detriment of women globally.

There is however hope that the tide may be turning following the recent publication of a report in the Lancet co-authored by multiple experts worldwide. Vogel et al. set out 10 key recommendations for reducing the global burden of CVD in women by 2030 [1]. They include recommendations to increase awareness and educate both healthcare professionals and the public, introduce large-scale heart health initiatives, and prioritize gender-specific research into women and the heart. 

Reassuringly the findings of this umbrella review undoubtedly echo the key points highlighted within this Lancet commission report [1] and increase hope that the outlook of women globally with and at risk of CVD will improve, and at last, the gender disparities and health inequalities seen within CV health will eventually become a factor of the past.

Strengths and limitations

Multiple databases were used in the initial search to minimise the possibility of missing relevant articles. This umbrella review includes the highest-level evidence from systematic reviews and meta-analyses, and following independent review using the GRADE scoring, nine of the 17 articles were deemed to be of high quality with this increasing to 14 with the inclusion of the moderate quality studies. Many of the included studies highlighted their own limitations due to lack of data, variability of research methods, and being limited to publishing in English increasing the chances of possible missed data.

The main limitation of this study is the lack of representation of females within the included studies. Although this is arguably the main weakness of this umbrella review, it also supports the key aim of it to analyse gender inequalities within CV health. Therefore, the lack of gender-based clinical data highlights the inability to progress in CV health until these inequalities are addressed. As noted, gender differences were first recognised in the early 2000s, however, we have not progressed forward since then.

All the included studies had a vast number of variables leading to high levels of heterogeneity, which was highlighted within many of the included articles individually. Generalisability may also currently be limited due to the articles reviewed being solely from Western countries and reported in English.

## Conclusions

The common themes highlighted within this umbrella review clearly demonstrate gender disparities to the detriment of women. Cardiovascular disease remains an equal-opportunity killer, yet the mortality burden remains higher in women than men. Although rates of mortality have improved with improved technology and developments in medicine, there remains a disparity with excess rates of mortality remaining in women due to under-recognition, under-diagnosis, and under-treatment. There is a need to prioritize women through raising awareness, improving prevention (both primary and secondary), reducing delays in presentation, and increasing understanding and recognition of sex differences in symptom presentation, enabling improved diagnosis and treatment along with standardisation of strategies to manage and follow up. It is clear within the included studies that further research is needed to understand the sex differences seen in both presentation and symptoms for cardiovascular disease and the need for further research to enable the development of gender-based clinical guidance has also been highlighted. Whatever developments are made through future research, they will undoubtedly contribute to reducing the gender-based inequalities currently apparent in cardiovascular health. 
